# Widely tunable two-colour seeded free-electron laser source for resonant-pump resonant-probe magnetic scattering

**DOI:** 10.1038/ncomms10343

**Published:** 2016-01-13

**Authors:** Eugenio Ferrari, Carlo Spezzani, Franck Fortuna, Renaud Delaunay, Franck Vidal, Ivaylo Nikolov, Paolo Cinquegrana, Bruno Diviacco, David Gauthier, Giuseppe Penco, Primož Rebernik Ribič, Eleonore Roussel, Marco Trovò, Jean-Baptiste Moussy, Tommaso Pincelli, Lounès Lounis, Michele Manfredda, Emanuele Pedersoli, Flavio Capotondi, Cristian Svetina, Nicola Mahne, Marco Zangrando, Lorenzo Raimondi, Alexander Demidovich, Luca Giannessi, Giovanni De Ninno, Miltcho Boyanov Danailov, Enrico Allaria, Maurizio Sacchi

**Affiliations:** 1 ELETTRA—Sincrotrone Trieste, Area Science Park, 34149 Trieste, Italy; 2 Dipartimento di Fisica, Università degli Studi di Trieste, 34127 Trieste, Italy; 3 Laboratoire de Physique des Solides, Université Paris-Sud, CNRS-UMR 8502, Bât. 510, 91405 Orsay, France; 4 Centre de Sciences Nucléaires et de Sciences de la Matière, Université Paris-Sud, CNRS UMR 8609, Bât. 104-108, 91405 Orsay, France; 5 Laboratoire de Chimie Physique Matière et Rayonnement, Sorbonne Universités, UPMC Univ Paris 06, CNRS UMR 7614, 75005 Paris, France; 6 Institut des NanoSciences de Paris, Sorbonne Universités, UPMC Univ Paris 06, CNRS UMR 7588, 75005 Paris, France; 7 Service de Physique de l'Etat Condensé, DSM/IRAMIS/SPEC, CNRS UMR 3680, CEA Saclay, 91191 Gif-sur-Yvette, France; 8 Dipartimento di Fisica, Università degli Studi di Milano, 20133 Milano, Italy; 9 Ecole Normale Supérieure, PSL Research University, 75231 Paris, France; 10 Graduate School of Nanotechnology, Università degli Studi di Trieste, 34127 Trieste, Italy; 11 Istituto Officina dei Materiali, Consiglio Nazionale delle Ricerche, 34149 Trieste, Italy; 12 ENEA, Centro Ricerche Frascati, Via E. Fermi 45, 00044 Frascati, Italy; 13 Laboratory of Quantum Optics, University of Nova Gorica, 5001 Nova Gorica, Slovenia; 14 Synchrotron SOLEIL, L'Orme des Merisiers, Saint-Aubin, B.P. 48, 91192 Gif-sur-Yvette, France

## Abstract

The advent of free-electron laser (FEL) sources delivering two synchronized pulses of different wavelengths (or colours) has made available a whole range of novel pump–probe experiments. This communication describes a major step forward using a new configuration of the FERMI FEL-seeded source to deliver two pulses with different wavelengths, each tunable independently over a broad spectral range with adjustable time delay. The FEL scheme makes use of two seed laser beams of different wavelengths and of a split radiator section to generate two extreme ultraviolet pulses from distinct portions of the same electron bunch. The tunability range of this new two-colour source meets the requirements of double-resonant FEL pump/FEL probe time-resolved studies. We demonstrate its performance in a proof-of-principle magnetic scattering experiment in Fe–Ni compounds, by tuning the FEL wavelengths to the Fe and Ni 3*p* resonances.

## Abstract (web summary)

Two-colour X-ray free electron laser is a powerful tool for pump–probe measurements, but currently constrained by limited tunability. Here, Ferrari *et al*. develop a configuration that allows tuning both the pump and the probe to specific electronic excitations, providing element selectivity.

## Graphical abstract

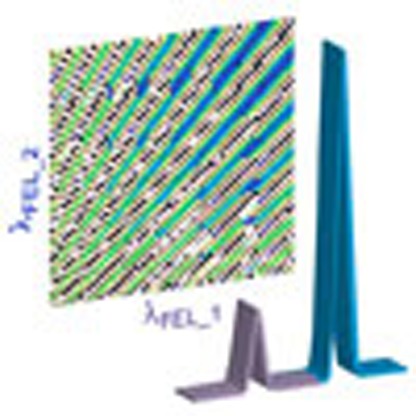

Free-electron laser (FEL) sources covering the wide spectral range from extreme ultraviolet to hard X-rays represent a breakthrough in photon science, with applications in physics, chemistry and biology. Many aspects of the spectral and temporal characteristics of the FEL pulses can be tailored to specific experimental needs by an accurate control of the lasing process, in the so-called beam by design approach^1^. The ability to run the FEL source in two-colour configuration, that is, to create two synchronized FEL pulses of differing wavelengths, has enormous potential for femtosecond time-resolved studies^2^,^3^ as it opens up unique opportunities for studying the dynamic response in atomic, molecular and solid state systems by selectively tuning electron resonances in atoms. As a consequence it has engendered major research^4^,^5^,^6^,^7^,^8^ and development^9^,^10^,^11^,^12^,^13^,^14^,^15^ efforts at all FEL facilities worldwide, with the ambition of attaining wide-ranging colour tunability and timing control.

Various two-colour schemes have been proposed, both for seeded^2^,^9^,^10^,^14^ and for self-amplified spontaneous emission (SASE)^11^,^12^,^13^ FEL sources. Initial configurations delivered two short FEL pulses with a controlled temporal separation in the range of a few hundred femtoseconds and a small photon wavelength separation (∼1%). Such configurations, where a single electron bunch generates the two FEL pulses, have served users for experiments both at seeded^2^ and at SASE^11^,^12^,^13^ facilities. In the case of SASE, differing photon wavelengths are obtained by dividing the radiator in two slightly detuned sections^11^. In the case of external seeding, the FEL wavelength separation is controlled by acting on the seed laser wavelength and by taking advantage of a residual controllable energy chirp on the electron beam^2^,^10^. For self-seeding schemes, it has been demonstrated^14^ that two seeded FEL pulses can be generated using two distinct Bragg diffraction lines in the self-seeding crystal recombined within the taper-tuned undulators. The possibility of producing two colours with a wider spectral separation (up to 30%) has been demonstrated recently at the SACLA hard X-ray SASE source by using the capabilities of a variable gap undulator^13^.

Until now, no configuration that generates two pulses with independently tunable wavelengths over a wide spectral range had been designed for externally seeded FELs. A whole new class of pump–probe experiments that require both pump and probe to be element selective is created by combining the full coherence of seeded FELs with a broad and independent tunability of the two colours.

Over the last decade, time-resolved studies made frequent use of short X-ray pulses as a probe that is coupled to an optical laser pump. Femto-slicing at synchrotrons^16^,^17^,^18^,^19^,^20^,^21^,^22^, high harmonic generation in gases^23^,^24^,^25^,^26^ and FEL sources^27^,^28^,^29^,^30^,^31^,^32^ deliver extreme ultraviolet and X-ray pulses with sub-100-femtoseconds duration that have been used for studying the ultrafast dynamics of magnetic^16^,^17^,^18^,^19^,^20^,^21^,^22^,^23^,^24^,^25^,^26^,^27^,^29^,^31^ and structural^20^,^28^,^29^,^30^ order in optical-laser pump/X-ray probe experiments. Tuning the wavelength to an atomic resonance provides the probe with element selectivity, which is of considerable interest especially for magnetic studies.

Developing FEL sources that can produce two pulses with independently selectable wavelengths for the pump and the probe and with a well-defined time separation obviously widens the potential of FEL radiation for studying the dynamics of a process and makes it possible to associate the pump energy to a specific electronic excitation of a given element. One field that will surely profit from this new tool is magnetization dynamics in 3*d*-transition-metal and rare-earth based oxides and compounds^18^,^19^,^22^,^33^,^34^,^35^,^36^,^37^: the presence of highly localized 3*d* and 4*f* orbitals and of mediated exchange interactions suggests that associating the pump energy to a specific electronic excitation will influence the magnetization dynamics profoundly, compared with using a non-resonant pump.

In the proof-of-principle time-resolved scattering experiment on Fe–Ni compounds described here, we use the new two-colour configuration of the externally seeded FERMI FEL source to generate, from the same electron bunch, two synchronized pulses with up to 30% spectral separation. The pump FEL pulse excites the Fe 3*p*→3*d* transition resonantly, while the second FEL pulse, tuned to the Ni 3*p*→3*d* resonance, probes the ultrafast Ni magnetization dynamics. The experiment successfully reveals the potential of this new source for investigating structural, electronic and magnetization dynamics in the fields of condensed matter as well as atomic and molecular physics.

## Results

### Two-colour seeded FEL with wide wavelength tunability

**Figure 1:**
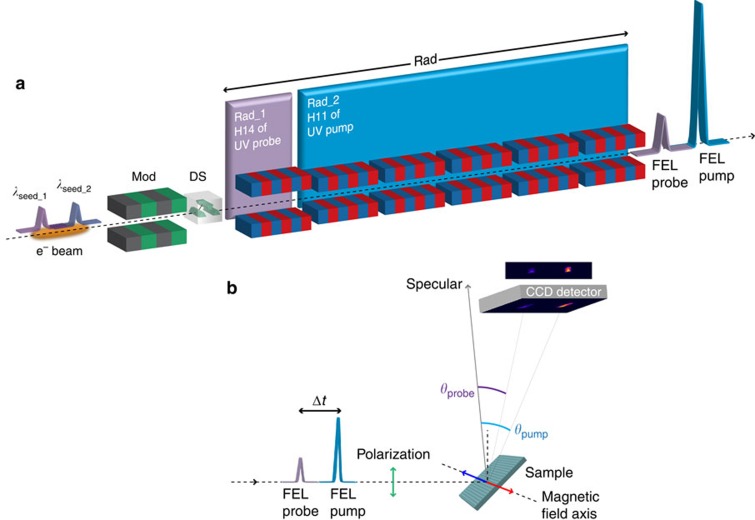
Schematic setup for a two-colour double resonance FEL experiment. (**a**) Two-colour seeded FEL source configuration: the modulator (Mod), dispersive (DS) and radiator (Rad) sections of the FEL source are outlined. In the modulator section, two ultraviolet (UV) laser pulses of wavelength *λ*_seed_1_ and *λ*_seed_2_ delayed by Δ*t* interact with the same electron bunch, imposing an energy modulation that is converted into density modulation in DS. The first radiator subsection Rad_1 is tuned to the 14th harmonic of *λ*_seed_1_ and the second subsection Rad_2 is tuned to the 11th harmonic of *λ*_seed_2_, generating the FEL probe and pump pulses, respectively (see Methods). (**b**) Magnetic scattering experiment: the two linear p-polarized FEL pulses reach the magnetic grating sample and diffract at different angles according to their wavelengths. The diffracted intensities are recorded by a two-dimensional detector (CCD camera). The wavelength separation between pump and probe is detected as a spatial separation at the CCD, while their time separation Δ*t* is defined by the delay between the two seed pulses.

The experiment was performed at the FERMI facility^38^,^39^, which is a seeded FEL operated in the high-gain harmonic generation (HGHG) mode^40^,^41^. The chosen configuration (see Methods) provided a relatively long (∼1 ps) electron bunch interacting with a short (∼100 fs) ultraviolet laser pulse (seed laser) in the first undulator section called the modulator (Mod in Fig. 1a). As a consequence of this interaction, the electron beam energy is modulated with a periodicity imposed by the seed laser wavelength *λ*_seed_. Following a magnetic chicane that works as a dispersive section (DS in Fig. 1a), the energy modulation is converted into a density modulation (bunching), which has strong harmonic components. Finally, in a second long undulator section called the radiator (Rad in Fig. 1a), the bunched electrons generate coherent FEL emission at one of the harmonics of the seed laser which is selected by setting the undulator gap. The advantages of HGHG with respect to SASE FEL stem from the fine control of the initial bunching, making it possible to generate FEL pulses with a high degree of longitudinal coherence^42^. Moreover, since only electrons interacting with the seed laser are bunched, this scheme provides a good control of the FEL temporal properties^43^.

Two FEL pulses with a controlled delay can be produced by seeding the same electron bunch with two seed pulses^2^. Since in the HGHG seeding process the final FEL wavelength is determined mainly by *λ*_seed_ and it must be close to one of its harmonics, a way for delivering two-colour FEL pulses with very different wavelengths (>10% separation) relies on seeding the electron beam with two laser pulses and on sustaining the amplification process at both wavelengths independently (Fig. 1a).

To achieve this, some constraints have to be dealt with. Both seed wavelengths *λ*_seed_1_ (for the probe) and *λ*_seed_2_ (for the pump) have to modulate the electron energy in the interaction region efficiently so their separation must be within the modulator working bandwidth. The two seed pulses modulate the energy in distinct regions of the electron beam. For each region, the dispersive section converts the energy modulation into an electron density modulation that carries all the harmonic components of the corresponding seed wavelength, either *λ*_seed_1_ or *λ*_seed_2_. The electron beam is now ready for the amplification of one of these harmonics, selected by the resonance condition of the radiator (undulator gap). A large separation between the two colours can be obtained by dividing the radiator into two subsections (Rad_1 and Rad_2 in Fig. 1a), one resonant at *λ*_FEL_1_=*λ*_seed_1_/*m* and the other at *λ*_FEL_2_=*λ*_seed_2_/*n*, with *m* and *n* integers. Since the radiator bandwidths are markedly narrower than the modulator one, we can emit efficiently the pump (or the probe) beam from one radiator subsection only, while suppressing its amplification in the other, selectively (see Methods). Finally, constraints on the temporal separation Δ*t* between the two FEL pulses are set by the need to avoid interference between the laser seeds (lower limit) and by the electron bunch duration (upper limit). In the example reported below, we spanned delays ranging from 300 to 800 fs.

The Fe-3*p* resonant-pump and Ni-3*p* resonant-probe test experiment (Fig. 1b) used two FEL pulses tuned to *λ*_FEL_2_=23.2 nm and *λ*_FEL_1_=18.7 nm, corresponding to the 11th harmonic of *λ*_seed_2_=255 nm and to the 14th harmonic of *λ*_seed_1_=261.5 nm, respectively. To this purpose, a special configuration of the FERMI seed laser was implemented based on the combined use of two ultraviolet pulses originating from a common infra-red source through two separated generation channels. One made use of an optical parametric amplifier (OPA) for producing the 255 nm seed, the other of a third harmonic generation (THG) setup for the 261.5 nm seed. This approach made the twin seeding possible at two different ultraviolet wavelengths, one of them tunable via the OPA (see Methods). We tested different distributions of the six undulator modules over the Rad_1 and Rad_2 radiator subsections (see Fig. 1a). We obtained different power distributions between pump and probe by going from one module in Rad_1 and five in Rad_2 to three modules in each subsection. All the configurations provided satisfactory stable conditions for producing two-colour FEL pulses. Since in our test experiment the pump is required to be more energetic than the probe, five of the six available radiator modules were tuned to produce 23.2 nm pulses (Rad_2 in Fig. 1a), while the remaining module (Rad_1) was tuned to the probe wavelength. It was important, in this configuration, that Rad_1 was the first of the undulator modules, to prevent the smearing of the electron density modulation along the radiator section to degrade its performance. We verified also that one can switch readily the FEL pump and probe wavelengths, by reversing the time delay between OPA and THG generated seed pulses and inverting the gap settings of the Rad_1 and Rad_2 radiator subsections.

**Figure 2:**
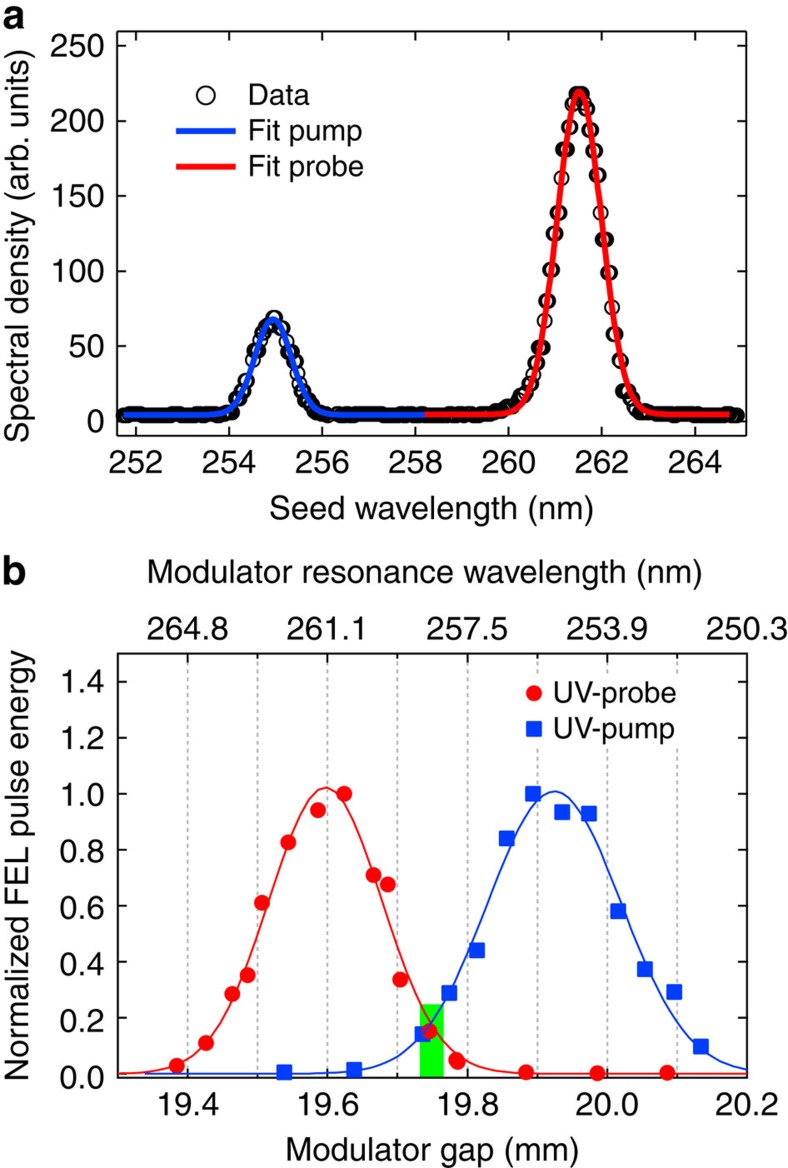
Seed pulses and modulator setting. (**a**) Spectral properties of the ultraviolet (UV) laser twin-seed source. Lines are Gaussian fits to the *λ*_seed_1_=261.5 nm (red line) and *λ*_seed_2_=255 nm (blue line) probe and pump contributions, respectively. (**b**) Modulator gap dependence of the FEL output for the two seed wavelengths. Circles and squares refer to seeding at 261.5 and 255 nm, respectively. Each point is the average of 100 consecutive FEL shots. Lines represent Gaussian fits to the intensity distributions. The curves are normalized to the same average maximum, showing that tuning the modulator gap to 19.75 mm (vertical green bar) makes it possible to seed with two colours simultaneously, preserving a fraction of the maximum pulse energy.

Figure 2a shows the spectral distribution of the two ultraviolet seed laser pulses and Fig. 2b shows the FEL pulse energy as a function of the modulator gap, when using only the ultraviolet -probe or only the ultraviolet -pump seeds. The two curves of Fig. 2b, which are normalized to the same amplitude, illustrate at each wavelength the extreme sensitivity of the FEL intensity to the modulator setting. A modulator gap of 19.94 mm optimizes the FEL pump emission when seeding at *λ*_seed_2_, while a gap of 19.60 mm is best when seeding at *λ*_seed_1_ to produce the FEL probe pulse. The gap can be used as an adjustable parameter for the fine control of the relative efficiency in the generation of the pump and probe FEL pulses, thanks to the ∼3% resonance bandwidth of the modulator (Supplementary Fig. 1). In our case, a good compromise was found at a gap of 19.75 mm, which made it possible to generate both *λ*_FEL_1_=18.7 nm and *λ*_FEL_2_=23.2 nm pulses, albeit with a reduced intensity. For the Fe–Ni experiment, the FERMI FEL source was characterized by pulse energies of up to ∼10 μJ at the pump wavelength and ∼1 μJ at the probe wavelength using these parameters. Once converted into a fluence *F* at the sample surface (see Methods), these values were sufficient to reach, in our experiment, the damage threshold and single shot detection conditions for the pump and the probe pulses, respectively.

### Resonant-pump/resonant-probe magnetic scattering experiment

**Figure 3:**
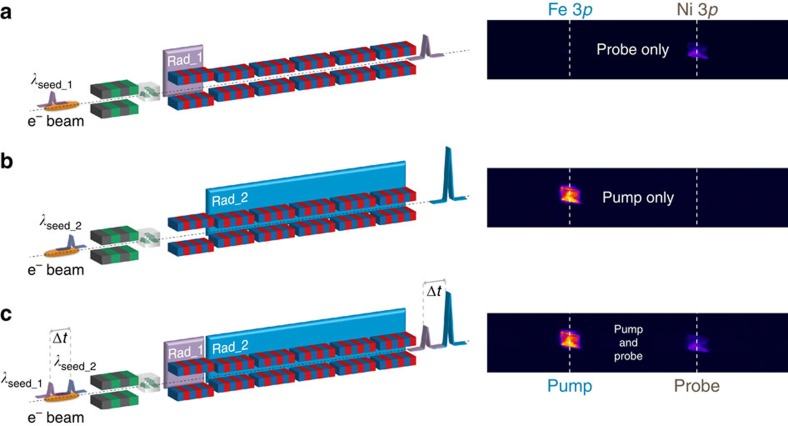
FEL source configuration and scattering data recording. Diffracted intensity from the 20-nm-thick permalloy grating sample at 46.3° incidence. Data are collected under different seeding conditions (schematics on the left) using a position-sensitive CCD detector (images on the right); the 1,025 × 202 pixel images correspond to 13.84 × 2.73 mm^2^ and cover ∼1.48° in scattering angle. (**a**) The *λ*_seed_1_=261.5 nm laser pulse is sent through the modulator, turning on the Ni-3*p* resonant FEL emission at *λ*_FEL_1_=18.7 nm in Rad_1 (14th harmonic) and no emission from Rad_2. (**b**) The *λ*_seed_2_=255 nm laser pulse generates the Fe-3*p* resonant FEL emission at *λ*_FEL_2_=23.2 nm in the radiator section Rad_2 (11th harmonic) and no emission from Rad_1. (**c**) Both seed laser pulses, delayed by Δ*t*, interact with the electron bunch, generating Fe-3*p* resonant pump and Ni-3*p* resonant probe FEL pulses, also delayed by Δ*t*.

We tested the two-colour twin-seeded FEL source by studying the resonant-pump/resonant-probe magnetization dynamics in Fe–Ni samples, using the IRMA reflectometer^44^ installed at the DiProI beamline^45^,^46^. The samples were a 20-nm-thick permalloy (Ni_0.81_Fe_0.19_ alloy) film deposited on a Si grating and a 12.5-nm-thick NiFe_2_O_4_ layer epitaxially grown on MgAl_2_O_4_(001). Both samples were structured as line gratings with a period of ∼600 nm (see Methods). They worked as dispersive elements, separating different wavelengths at the level of the two-dimensional in-vacuum charge-coupled device (CCD) detector^23^,^24^. All Bragg peaks generated by the grating samples at different wavelengths fell within the angular acceptance of the detector and could be collected simultaneously (see Fig. 3).

**Figure 4:**
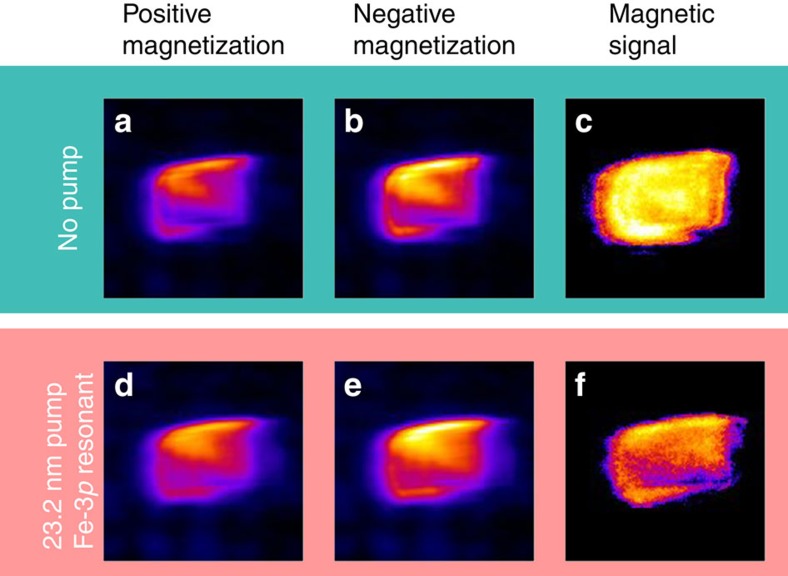
Magnetic signal in the scattering data. Diffracted intensity at the Ni-3*p* resonant probe wavelength with no pump (**a**–**c**) and following a Fe-3*p* resonant pump pulse (**d**–**f**). The pump fluence is *F*=8 mJ cm^−2^, the delay Δ*t* is 450 fs. (**a**,**d**) and (**b**,**e**) Diagrams refer to a positive and negative saturating magnetic field, respectively. The magnetic signal, expressed as the asymmetry ratio, is shown in **c** and **f**. Each picture is 128 × 128 pixels, corresponding to 1.73 × 1.73 mm^2^.

The FEL polarization was set to linear vertical to optimize the sensitivity to the sample magnetization in transverse geometry^23^,^24^,^47^,^48^,^49^, that is, with the external magnetic field applied normal to the scattering plane and parallel to the lines of the grating sample (see Fig. 1b). After an initial 80 mT magnetic pulse, the scattered intensity was collected in an applied field of 20 mT, guaranteeing the sample magnetic saturation (see Methods). In the following, the magnetic signal is defined as an asymmetry ratio, that is, as the difference between scattered intensities measured for opposite signs of the applied field divided by their sum, as shown in Fig. 4. At each given delay Δ*t*, the Ni magnetic signal was measured as a function of the pump fluence *F* (see Methods for the relationship between FEL pulse energy and fluence at the sample). The pump wavelength was tuned either to the Fe-3*p* resonance (*λ*_FEL_2_=23.2 nm) or off-resonance (*λ*_FEL_2_=25.5 nm), the latter being obtained simply by tuning the radiator subsection Rad_2 to the 10th harmonic of the λ_seed_2_ seed laser wavelength, instead of the 11th. It is worth underlining that, according to calculations based on tabulated optical constants^50^ (see also http://henke.lbl.gov/optical_constants/), the fraction of pump energy absorbed by the sample at 23.2 nm and at 25.5 nm differs by less than 2% for both permalloy and ferrite films.

**Figure 5:**
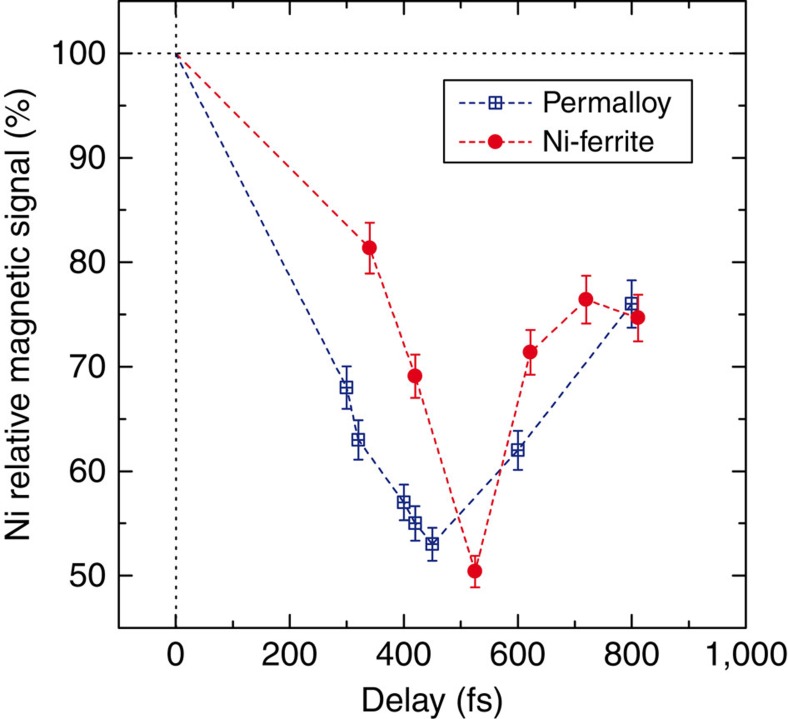
Time-dependent magnetic signal. Ni demagnetization in the permalloy (blue squares) and the Ni-ferrite (red circles) samples at several delays Δ*t* between probe and pump pulses (*F*=10 mJ cm^−2^). Vertical error bars represent s.d. (see Methods). The maximum fluctuation in the pump–probe delay over the measurement duration (±5 fs, see Methods) is smaller than the point width. Lines are a guide to the eye.

First, we explored the ultrafast Ni demagnetization while varying the delay Δ*t* between the FEL probe and pump by adjusting the delay between the corresponding seed laser pulses. An example of delay dependence spanning the 300–800 fs range is shown in Fig. 5 where the Ni magnetic signal is reported after a Fe-3*p* resonant pump pulse with fluence *F*=10 mJ cm^−2^ (dots and squares refer to Ni-ferrite and permalloy samples, respectively). The asymmetry ratio in the Bragg peak intensity is calculated over a limited detector area of ∼100 × 100 μm^2^ to ensure homogeneous pump fluence and the Ni magnetic signal is normalized to its static value measured with no pump.

**Figure 6:**
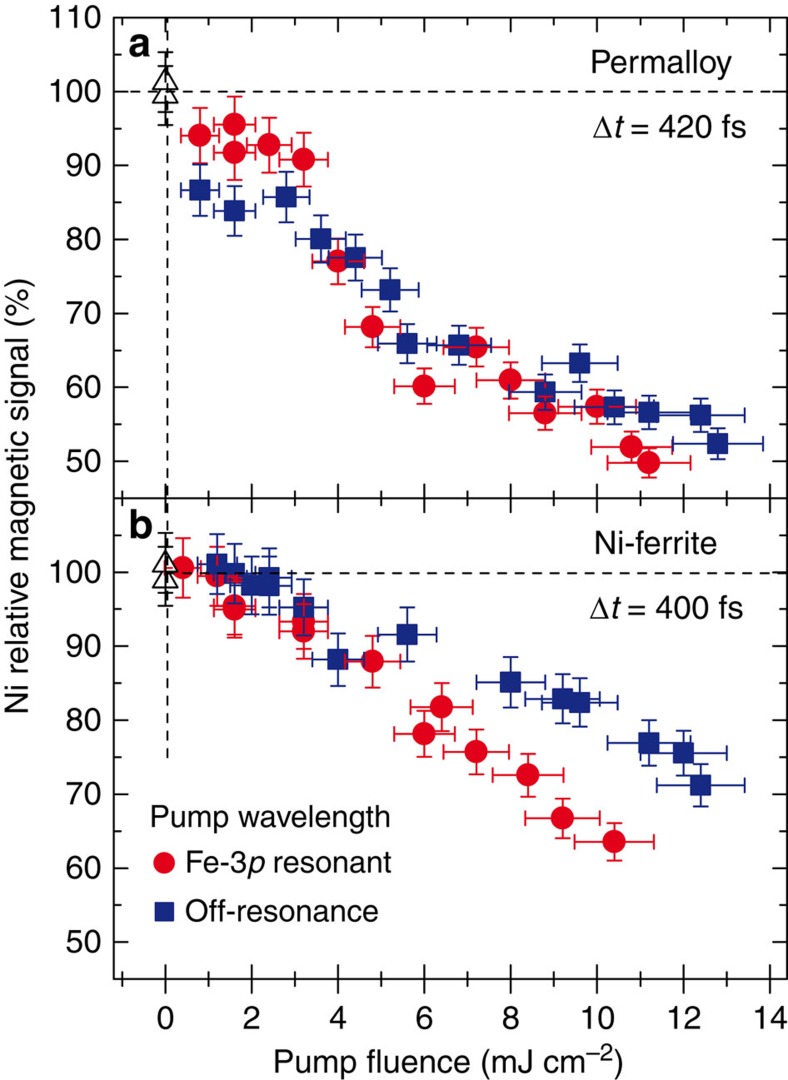
Resonant versus non-resonant pumping. Pump fluence dependence of the Ni demagnetization in permalloy (**a**) and Ni-ferrite (**b**) at ∼400 fs delay, comparing the results for Fe-3*p* resonant (*λ*_FEL_2_=23.2 nm, red circles) and non-resonant (*λ*_FEL_2_=25.5 nm, blue squares) FEL pump pulses. The Ni magnetic signal is reported as the asymmetry ratio in the Bragg peak intensity, normalized to the value measured with no pump (open triangles). Error bars represent s.d. (see Methods).

The main advantage of this novel two-colour scheme over those developed previously at the FERMI seeded source^2^ is its ability to tune both *λ*_FEL_1_ and *λ*_FEL_2_ to selected values over a broad range. It is also important to stress that this scheme makes the switching between on- and off-resonance pumping fast and easy. As mentioned before, this can be achieved simply by changing the gap of the Rad_2 radiator subsection for selecting a different harmonic of the *λ*_seed_2_ wavelength. An example of on/off-resonance pumping is given in Fig. 6. It shows the Ni magnetic signal (normalized to its static value) measured at a fixed time delay of ∼400 fs for a FEL pump wavelength tuned to the Fe-3*p* resonance (*λ*_FEL_2_=23.2 nm, red circles) or off-resonance (*λ*_FEL_2_=25.5 nm, blue squares) as a function of the pump fluence *F*. The permalloy results (Fig. 6a) do not reveal a measurable effect of the pump wavelength: both curves show the same *F*-dependence of the Ni magnetic signal, which attains a ∼50% reduction at *F*∼10 mJ cm^−2^. On the contrary, pumping at the two on/off-resonance wavelengths results in an apparent difference in Ni demagnetization behaviour when *F* exceeds ∼5 mJ cm^−2^ in the case of Ni-ferrite (Fig. 6b).

Although a detailed discussion of the results reported in Figs 5 and 6 is not within the scope of this communication, the observed differences between ferrite and permalloy behaviour can be ascribed to the direct hybridization of delocalized Fe and Ni 3*d* orbitals in ferromagnetic permalloy versus indirect exchange (via oxygen) of more localized 3*d* orbitals in ferrimagnetic NiFe_2_O_4_. These early results are intriguing and more studies are under consideration to shed light on the observed pump wavelength dependence.

## Discussion

We have developed and tested a new FEL setup capable of delivering two-colour time-delayed pulses with independent wavelength tunability over a wide spectral range (18.7–25.5 nm). Combined with the seeded nature of the FERMI source^39^, this provides improved conditions for two-colour FEL experiments that require tuning both the pump and the probe to selected atomic resonances. The potential of this two-colour scheme has been demonstrated by a scattering experiment that probes the magnetization dynamics in systems containing two magnetic elements, Fe and Ni. Undoubtedly, it can find original applications in many other fields of condensed matter, atomic and molecular physics.

From a technical point of view, the solution that we propose is based on seeding the same electron bunch with two independent laser pulses and on splitting the FEL radiator into two subsections. On one hand, this solution offers the possibility of selectively tuning the two FEL colours over a very wide range. It may go well beyond the 30% bandwidth demonstrated here, by amplifying different harmonics of the seed wavelengths in each radiator subsection. On the other hand, using two laser seeds that modulate the same electron bunch, and two radiators impose some constraints on the relationship between the *λ*_FEL_1_ and *λ*_FEL_2_ wavelengths, both in terms of FEL intensity and of possible gaps in the range of wavelengths that can be spanned.

**Figure 7:**
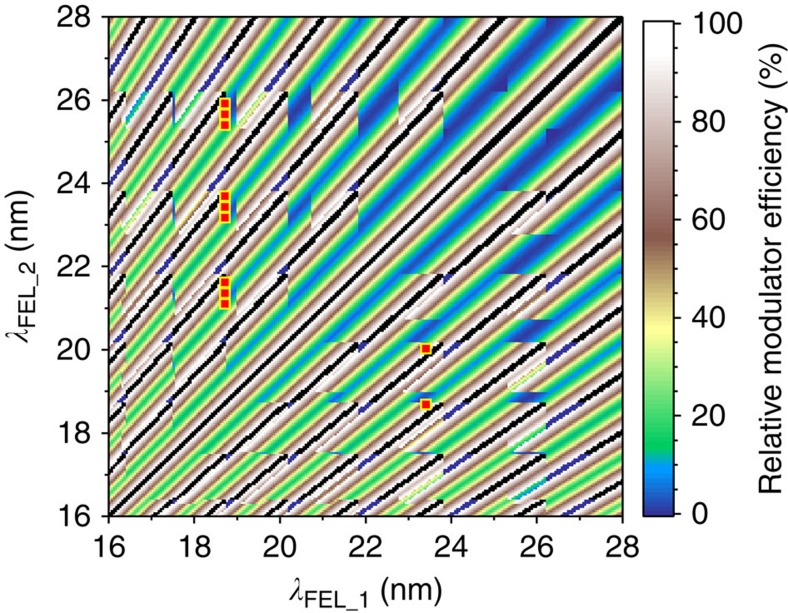
Calculated seeding efficiency over the 16–28 nm range. The colour code represents the relative modulator efficiency at *λ*_FEL_1_ and *λ*_FEL_2_ when the modulator gap is set to resonate with their average value. The calculation uses radiator harmonics from 9 to 16 and *λ*_seed_ values between 228 and 262 nm. Black dots correspond to (*λ*_FEL_1_, *λ*_FEL_2_) couples whose *λ*_seed_ values are within the radiator bandwidth and cannot be produced using the proposed source scheme. Red squares identify the couples of wavelengths explored during the test experiment (Supplementary Figs 2 and 3).

Figure 7 summarizes the calculated source performance when *λ*_FEL_1_ and *λ*_FEL_2_ span the 16–28 nm range, showing that marked intensity variations are present. The colour code represents the relative modulator efficiency for each couple of wavelengths, calculated assuming that the modulator resonance is set to the average value of λ_FEL_1_ and λ_FEL_2_. Both seed wavelengths are allowed to span the 228–262 nm range covered by the OPA, and radiator harmonics from 9 to 16 are considered. The finite modulator bandwidth defines the maximum intensity that can be obtained for each (*λ*_FEL_1_, *λ*_FEL_2_) combination, hence the efficiency of the two-colour process. The radiator bandwidth imposes limitations on the independent tunability of *λ*_FEL_1_ and *λ*_FEL_2_: black dots forming diagonal lines in Fig. 7 mark couples of wavelengths whose corresponding *λ*_seed_ values are close enough to be amplified in both radiator subsections. In this case, four FEL pulses, and not two, would be generated and the proposed two-colour scheme does not work properly.

Figure 7 shows the tuning capabilities and limitations of the adopted two-colour FEL scheme over the 16–28 nm range that broadly covers the wavelengths used in our test experiment. The red squares in Fig. 7 indicate the pairs of FEL wavelengths that were actually explored for the Fe–Ni double-resonant pump–probe measurements (Supplementary Fig. 2 and Supplementary Fig. 3). In principle, a much wider range of *λ*_FEL_ values extending up to 90 nm can be covered by using the full set of harmonics available at FERMI^39^,^51^. At wavelengths longer than ∼45 nm, though, the limited range of the OPA and the low radiator harmonic numbers introduce gaps in the (*λ*_FEL_1_, *λ*_FEL_2_) values that can be covered by this two-colour FEL scheme (Supplementary Fig. 4).

The accessible delay range between the pump and the probe is limited by the generation of the two FEL pulses from the same electron bunch of finite temporal length. In our experiment, we spanned the 300–800 fs range and an extension to 200–1,000 fs can be envisaged. This remains a strong constraint on the class of dynamic phenomena that can be addressed. Concerning ultra-fast demagnetization, in particular, many systems of interest feature response times of the order of 200 fs (refs 21, 26, 52), at the limit of the accessible range.

Further developments can be envisaged for improving the source characteristics, such as the twin-bunch mode recently demonstrated in SASE configuration^15^. The implementation of a similar scheme at FERMI would provide a more efficient bunching at the two wavelengths, a more efficient coupling in the radiator sections and, in fine, a significant increase in the energy per pulse, which could attain tens of microjoules for both the pump and the probe. Moreover, using two independent bunches would provide additional flexibility for tuning the two *λ*_seed_ wavelengths and would soften the constraints on the temporal separation between pump and probe pulses. Another significant improvement, already planned at FERMI, implies a second OPA for tuning both *λ*_seed_ wavelengths independently, as used for computing the tuning range reported in Fig. 7 and Supplementary Fig. 4. The desired resonant condition for both the pump and the probe FEL pulses could be finely matched.

Finally, it is worth remembering that the FERMI radiator section is composed of Apple-II type undulators^53^ delivering radiation of selectable polarization, either circular (right/left) or linear (vertical/horizontal). Therefore our two-colour source offers the possibility of choosing the polarization state of each pulse independently, which may be especially important in atomic and molecular physics studies.

The two-colour extreme ultraviolet source that we have developed at FERMI already has potential for many interesting and original studies in magnetization dynamics and beyond. For instance, it can cover the 3*p* resonances of any couple of elements among Mn, Fe, Co and Ni, making a wide class of relevant magnetic materials accessible to resonant FEL pump/resonant FEL probe experiments. More generally, it enables the excitation of a particular energy and polarization-selected resonance on a well-defined atomic site in a complex system and makes it possible to study its dynamics with the second FEL pulse, by choosing for the probe another electronic subshell or another atomic site. This new source will provide unprecedented opportunities for probing in a highly selective way the dynamics of complex relaxation processes, such as Auger cascades or sequential multiple ionization, and of charge transfer processes in large molecules and clusters.

## Methods

### Accelerator

The FERMI linac^54^ was operated at 1.3 GeV electron beam energy and 700 pC nominal charge. A moderate compression produced almost flat 500 A current electron bunches. The bunch length provided the conditions for an effective twin seeding with temporal separation of up to ∼900 fs. The longitudinal phase space of the electron beam (energy versus time) was characterized by a chirp with both linear and quadratic components^55^ that can be exploited to further enhance the difference between the resonant wavelengths in the two parts of the beam.

### Seed lasers

The special twin-seed laser configuration for wide tunability two-colour FEL was based on the standard FERMI seed laser system described earlier^56^,^57^,^58^. The output of a Ti:Sapphire amplifier (5–7 mJ per pulse, 100 fs pulse duration, 784 nm central wavelength) was shared between an infrared OPA and a THG setup. Inside the OPA box, the signal pulses delivered by a two-stage white-light-seeded OPA process were frequency mixed with a residual pump pulse and the generated visible light was further up-converted by second harmonic generation to obtain ultraviolet pulses in the 228–262 nm range. The second ultraviolet pulse, generated in a time-plate-type BBO crystal-based THG setup, was adjusted to generate pulses with a central wavelength of 261.5 nm. The intensities of the two pulses could be varied independently through remotely controlled waveplates. The time delay between the two seed pulses was measured using an optical cross-correlator, where each ultraviolet pulse was cross-correlated with an IR pulse derived from the ultrafast oscillator that seeds the Ti:Sapphire amplifier. A remotely controlled delay stage on the THG path was used to set the time delay between the two seed pulses, before recombining them through a 50% beam splitter. Both seed pulses originate from the same source (laser oscillator and regenerative amplifier) and their relative time delay is very stable^56^,^57^. It has been verified that once set, the relative time delay between the two ultraviolet pulses (hence between the two FEL pulses) remains stable within less than ±5 fs over a time span of 2 h. This includes both short-term timing jitter and slow timing drifts. The adjustment and long-term stabilization of the spatial coincidence and collinearity of the two seed beams inside the FEL undulator, which are essential for obtaining the coincidence of the two FEL pulses on the sample, were obtained by using a dedicated feedback loop based on independent steering optics for each beam.

### Undulators

The modulator is a 100 mm period 3 m long planar undulator with ∼3% nominal resonance bandwidth. The radiator comprises six independent 55 mm period 2.42 m long undulators based on the APPLE–II design^53^ that provide adjustable polarization^59^,^60^. The radiator was divided into two subsections, Rad_1 and Rad_2, set to resonate with harmonic 14 of *λ*_seed_1_ and harmonic 11 of *λ*_seed_2_, respectively (Fig. 1a). The *λ*_seed_2_=255 nm OPA seed produces a localized bunching at all the harmonics including the 11th that matches the resonance in Rad_2, generating 23.2 nm coherent emission which is amplified along the radiator. However, the beam has also bunching at the 18.2 nm 14th harmonic close to the resonant wavelength of Rad_1 (18.7 nm), which may produce unwanted emission. Similarly, the *λ*_seed_1_=261.5 nm THG seed induces a bunching at 18.7 nm (14th harmonic), which generates the FEL probe pulse in Rad_1, but also at 23.8 nm (11th harmonic) which may excite emission from Rad_2 tuned at 23.2 nm. In both cases, though, the separation between the undesired bunching wavelength and the radiator resonant wavelength is >2%, that is, larger than the ∼0.7% gain bandwidth measured for the radiators (Allaria *et al*. FEL-1 current status and recent achievements, FERMI Machine Advisory Committee, Sincrotrone Trieste, April 2014, unpublished). It is the narrow bandwidth of the radiators compared with the modulator that makes it possible to produce time-delayed single-frequency pump and probe FEL pulses from the same electron bunch.

### Samples

The 20 nm permalloy film was sputter-deposited from a Fe_19_Ni_81_ target onto a commercial Si grating (605 nm period, 190 nm groove depth), with 3 nm Al buffer and capping layers. Room temperature magneto-optical Kerr effect measurements showed 100% remanence and ∼8 mT coercive field along the grating lines. The 12.5-nm-thick NiFe_2_O_4_ layer was grown on MgAl_2_O_4_(001) by molecular beam epitaxy in atomic oxygen plasma. A 100 × 400 μm^2^ area of the Ni-ferrite layer was ruled by focused ion beam etching with a set of ∼350 nm wide stripes with a ∼600 nm period. The magnetic signal at the Fe-3*p* resonance measured on the patterned area at the FEL source showed an ∼50 mT coercive field with 100% remanence along the stripes.

### Scattering setup

The experiment was performed using the IRMA vertical-scattering-plane reflectometer^44^. A horseshoe electromagnet applied variable fields (±150 mT) parallel to the sample surface and normal to the scattering plane (Fig. 1b). The FEL beam was refocused at the sample position by two bendable mirrors in Kirkpatrick–Baez configuration, using an extreme ultraviolet imager at the sample position. The final spot size (∼80 μm) was estimated by scanning a movable pin-hole while measuring the transmitted intensity. The reflectometer allowed for a precise alignment of the sample with respect to the FEL beam using a slitted photodiode mounted on the detector arm. The vertically scattered intensity was detected by an in-vacuum CCD camera (2,048 × 2,048 pixels, pixel size 13.5 × 13.5 μm^2^) shielded from visible light by a 100-nm-thick Al filter. The CCD was mounted at 90° from the incoming FEL beam and at 535 mm from the sample. The pump fluence *F* at the sample was evaluated by correcting the pump energy measured at the source for the transport-line transmission (six reflections and a 200-nm-thick Al filter), focal spot size (∼80 × 80 μm^2^) and angle of incidence (46.5°). Error bars on fluence (Fig. 6) account for both the pump energy measurement accuracy and for the source intensity fluctuations. The maximum fluence at the sample was ∼40 and ∼3.5 mJ cm^−2^ for the pump and the probe, respectively. *F* values could be adjusted rapidly and continuously by attenuating the pump seed laser. The scattering of the p-polarized FEL radiation was measured near the Brewster extinction condition, reducing non-magnetic contributions and maximizing the magnetic contrast^23^,^24^,^47^,^48^,^49^. All the data reported here were collected at 46.5° incidence of the FEL radiation. Magnetization-dependent data were collected following the same protocol for both samples and for all the measurements: (a) application of +80 mT pulse of ∼10 ms duration, exceeding the saturation field; (b) +20 mT applied while collecting the scattered intensity at the CCD detector during a given acquisition time (1–10 s per frame); (c) repeat (a,b) for negative field values; (d) repeat the whole (a–c) sequence 50 times. The magnetic signal was then defined as an asymmetry ratio, that is, as the difference divided by the sum of two images collected for opposite signs of the applied field. Data reported in Figs 5 and 6 represent average values taken over a 7 × 7 pixels area.

## Additional information

**How to cite this article:** Ferrari, E. *et al*. Widely tunable two-colour seeded free-electron laser source for resonant-pump resonant-probe magnetic scattering. *Nat. Commun.* 7:10343 doi: 10.1038/ncomms10343 (2016).

## Supplementary Material

Supplementary Information Supplementary Figures 1-4
