# Supplementary material for: Widely tunable two-colour seeded free-electron laser source for resonant-pump resonant-probe magnetic scattering
Source: Nat Commun. 2016 Jan 13;7:10343. doi: 10.1038/ncomms10343 (PMC4735510; doi:10.1038/ncomms10343)
Supplement: Supplementary Information — Supplementary Figures 1-4 [file ncomms10343-s1.pdf]

Supplementary Figures

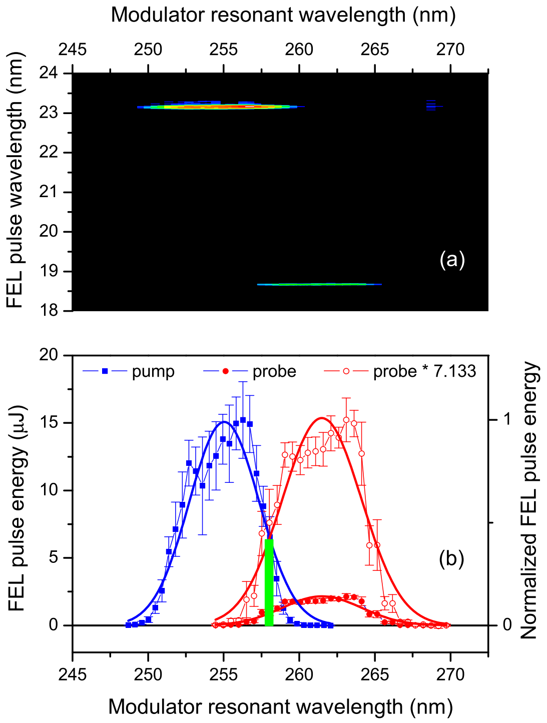

Supplementary Figure 1 | FEL pulse energy as a function of the modulator resonant wavelength. (a) Spectral analysis of the two-colour FEL emission using the FERMI in-line spectrometer. Data are shown as a function of the resonant wavelength defined by the modulator gap. **(b)** FEL pulse energy with only the pump (squares) or the probe (filled circles) laser seed. Normalizing the probe FEL energy curve to that of the pump by a multiplication factor (hollow circles) highlights the modulator setting that optimizes the emission at both FEL wavelengths. Data in panel **(b)** are similar to those of Fig. 2b, but collected during a later run with a different optimization of the machine parameters. In particular, the dispersive section magnetic field (R56 parameter) is stronger with respect to measurements shown in Fig. 2b, producing saturation for the on-resonance modulator setting. The simultaneous interaction with both seed pulses requires the modulator to be operated off-resonance and implies a loss in the seeding efficiency at both wavelengths. Operating with a stronger R56 (i.e., in over-bunching conditions with respect to optimal seeding) can compensate partly this loss, since the FEL response *vs.* the modulator gap differs from a typical Gaussian lineshape and can increase the effective bandwidth of the modulator, ensuring a better overlap of the emission at the two wavelengths (vertical green bar). The use of a stronger dispersion may be useful for optimizing configurations that require a spectral separation between the two seed pulses close to or beyond the modulator bandwidth.

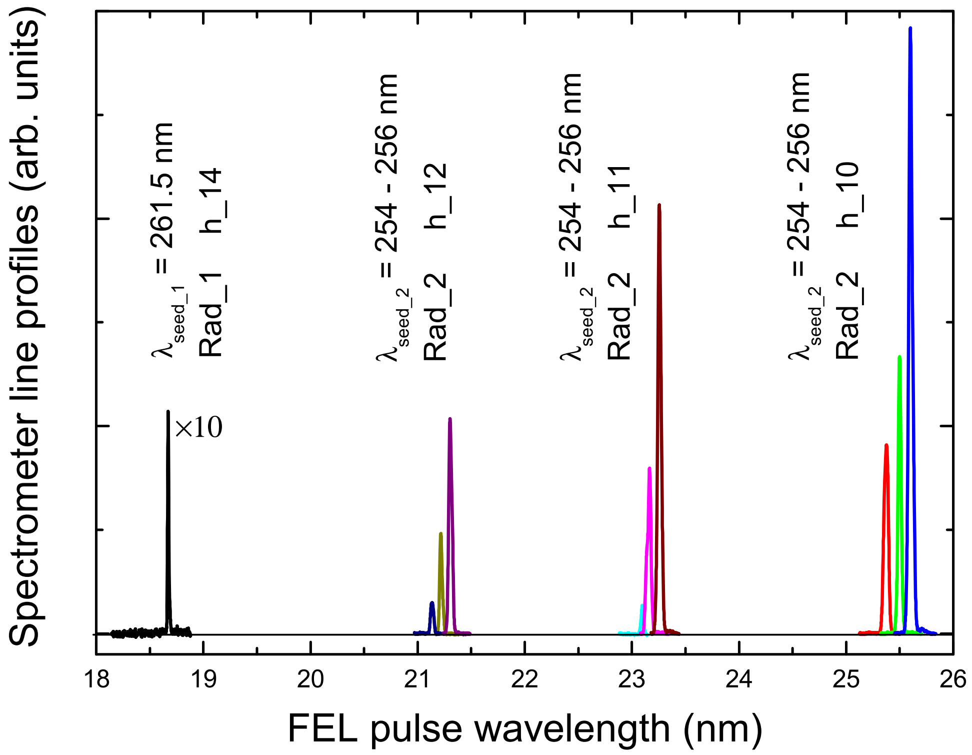

Supplementary Figure 2 | Varying the pump wavelength of the two-colour FEL source. Two-colour spectra collected using the FERMI in-line spectrometer. The spectra illustrate examples of changing the pump wavelength by either tuning the Rad_2 pump radiator subsection to different harmonics or by changing the pump UV seeding wavelength λ_{seed_2} via the optical parametric amplifier. All data are reported on a consistent scale of arbitrary units. The probe seed wavelength is λ_{seed_1} = 261.5 nm and Rad_1 is set to harmonic h_14, generating the Ni-3p resonant λ_{FEL_1} = 18.7 nm FEL probe pulse. The pump FEL wavelength is varied by setting λ_{seed_2} to three different values (254, 255 and 256 nm) and by changing the Rad_2 gap to resonate with harmonics h_10, h_11 and h_12. When Rad_2 is set to h_11, the pump is tuned to the Fe-3*p* resonance (23.1–23.4 nm range) while off resonance pumping is obtained by setting h_10 (~21.3 nm) and h_12 (~25.5 nm). The UV seed intensity is kept constant for all spectra and the FEL intensity variations reflect the efficiency of the two-colour process at different wavelength combinations.

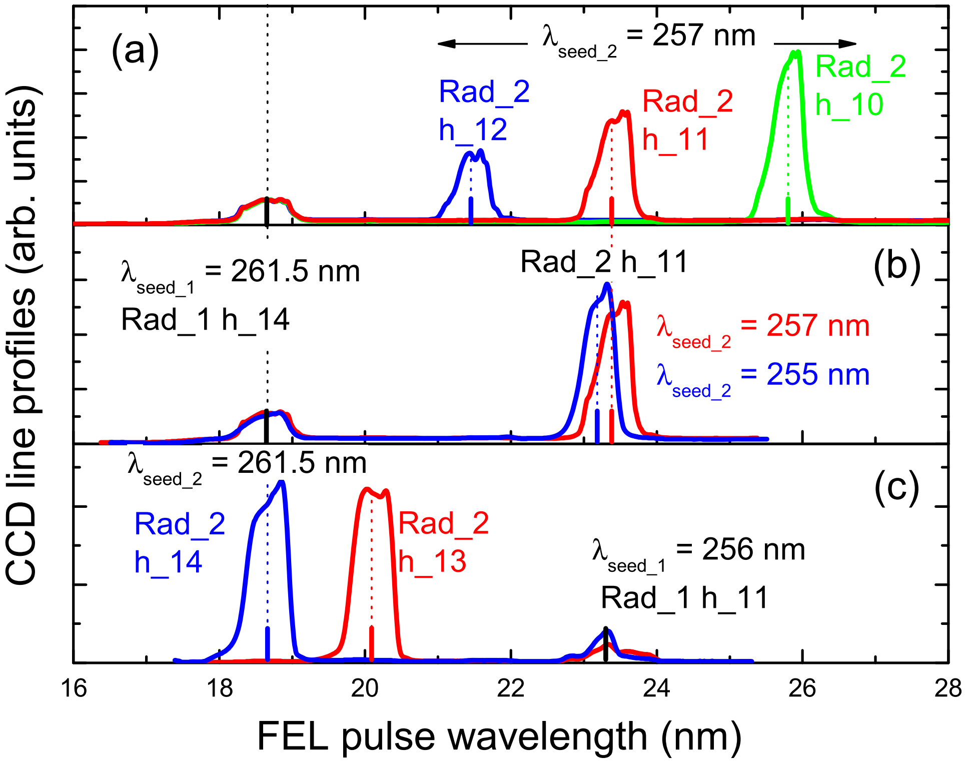

Supplementary Figure 3 | Different configurations of the two-colour FEL source. Two-colour spectra collected using the scattering setup described in the main text, after converting the CCD detector pixel number into FEL radiation wavelength. As in Supplementary Fig. 2, the spectra illustrate examples of changing the FEL wavelength by either tuning the radiator subsection to a different harmonic or by changing the UV seeding wavelength via the optical parametric amplifier. The FEL probe is delayed by 500 fs with respect to the pump. (**a**) Spectra obtained using λ_{seed_1} = 261.5 nm and Rad_1 set to h_14 for the probe pulse. Using λ_{seed_2} = 257 nm, the pump FEL radiation is tuned to three different wavelengths by selecting h_10 (25.7 nm), h_11 (23.4 nm) and h_12 (21.4 nm) in Rad_2. (**b**) Finer tuning across the Fe-3*p* resonance is obtained by adjusting λ_{seed_2} (255 and 257 nm are shown) with Rad_2 set to h_11. In panels (**a**, **b**) the probe wavelength is fixed to the Ni-3*p* resonance (18.7 nm) and the pump is tuned either to the Fe-3*p* resonance (23.2–23.4 nm) or off-resonance (21.4 and 25.7 nm). Panel (**c**) illustrates the reversed pump-probe wavelength scheme, with the one-module Rad_1 radiator sub-section set to the 11th harmonic of λ_{seed_1} = 256 nm (Fe-3*p* resonance) and the five-module Rad_2 sub-section set to either the 14th (Ni-3*p* resonance) or 13th (off-resonance) harmonic of λ_{seed_2} = 261.5 nm.

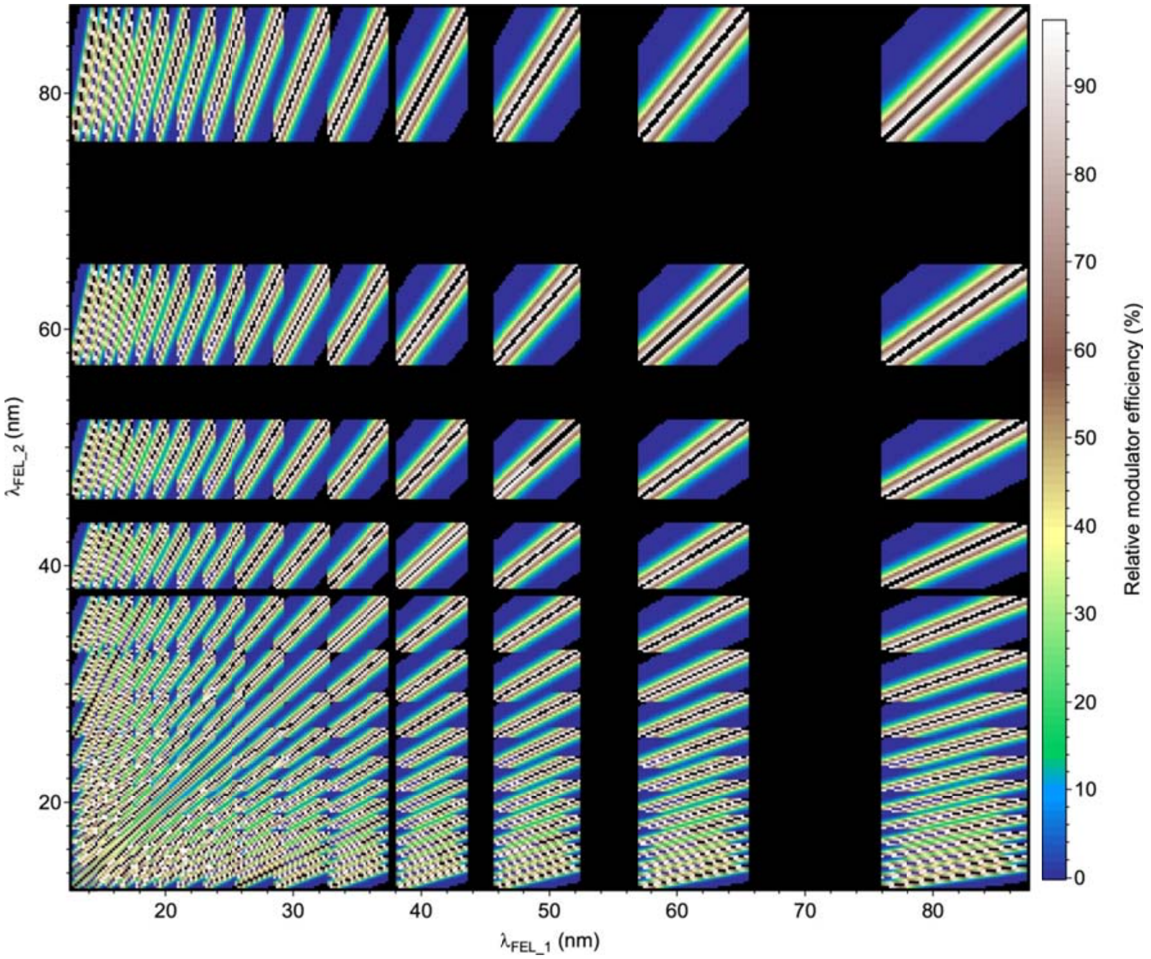

Supplementary Figure 4 | Calculated seeding efficiency over the 12-88 nm range. The colour code represents the relative modulator efficiency at λ_{FEL_1} and λ_{FEL_2} when the modulator gap is set to resonate with their average value. Calculations make use of radiator harmonics from 3 to 18 and λ_{seed} values span the 228 and 262 nm range. Black areas around 45, 55 and 70 nm correspond to λ_{FEL} values that cannot be covered by the FERMI FEL source using the λ_{seed} range given above. Black dots forming diagonal lines identify (λ_{FEL_1} , λ_{FEL_2}) couples whose corresponding λ_{seed} values are within the radiator bandwidth and cannot be produced as two-colour FEL pulses using the proposed source scheme. Dark blue areas correspond to (λ_{FEL_1} , λ_{FEL_2}) couples that can be produced, but with an intensity that is only a few percent of that obtained with the optimal modulator setting.
